# "Spontaneous scar dehiscence of a repaired bladder rupture in a 5 yr old girl – a case study"

**DOI:** 10.1186/1757-1626-1-363

**Published:** 2008-12-01

**Authors:** Abhijit Singh

**Affiliations:** 1 Resident Medical Officer, Max Heart and Vascular Institute, Saket, New Delhi, India

## Abstract

Spontaneous scar dehiscence of a previous repaired urinary bladder rupture is described in a 5 yr old girl approximately 4 yrs after the original repair, she presented at the hospital with the chief complain of pain and distension of the abdomen. No underlying aetiology could be related to the event, her blood urea and creatinine levels were found to be raised. A diagnostic peritoneal tap showed non bloody straw colour urine in the peritoneal cavity. Emergent laparotomy was carried out and the site of scar dehiscence was closed in layers.

## Introduction

Delayed scar dehiscence in bladder rupture is rare unlike that of the uterine dehiscence which is seen more commonly in association with some underlying pathology or in some cases may be iatrogenic following radiotherapy. But spontaneous or idiopathic bladder scar dehiscence is even more uncommon, only 2 cased being documented. I present a case of spontaneous scar dehiscence of a repaired bladder rupture after 4 yrs in a 5 yr old girl in Delhi.

## Case presentation

Presenting complain – A 5 yr old Indian girl presented at the hospital with the chief complain of pain and distension of the abdomen. She complained of pain for last 4 days, the pain was continuous in nature and diffusely involved the whole abdomen but was more so in the umbilical and the hypo-gastric regions, pain was non-radiating, was relieved on lying still and aggravated on movement. No predisposing cause of the pain could be found and the girl's mother said that she complained of stomach pain suddenly. The pain had gradually increased in intensity over time. Abdominal distension developed about 2 days after she complained of pain. She also complained of multiple episodes of vomiting (about 15 per day), the vomitus was greenish yellow in colour, non bloody, odourless and copious in amount. She complained of burning micturation as well and described an increase in pain during urination. She also complained of loss of appetite and mild constipation but denied any fever or cough. Past history – Her mother described that she had developed similar kind of features 4 yrs back when a ceiling fan had fallen on her stomach, she was hospitalized and was diagnosed as a case of intrauterine rupture of urinary bladder. Exploratory laparotomy was performed and bladder rupture was repaired. The post operative recovery was uneventful with no further complains.

Birth history – She was a full term normal spontaneous vaginal delivery, her development milestones and vaccination status are up to date.

### On examination

The girl was conscious and alert and was in pain.

Height-103 cms, Weight-28 kgs

Vital signs as follows: temp-98.8^F, pulse-126/min, RR-28/min, BP-100/70 mm Hg.

Abdominal exam-previous operative scar, abdomen distended, diffuse tenderness over whole abdomen, no rigidity, no guarding, liver and spleen impalpable, shifting dullness and fluid thrill were found to be present, bowel sounds were absent.

Examination of rest of the systems was uneventful.

### Investigations

Hb-12.5 gm%, TLC-14000, Na-130 mEq/L, K-6.5, Urea-210 mg/dl, creatinine-2.4 mg/dl, bilirubin-0.6(direct-0.3)mg/dl, AST-85 iu, ALT-49 iu.

**Figure 1:**
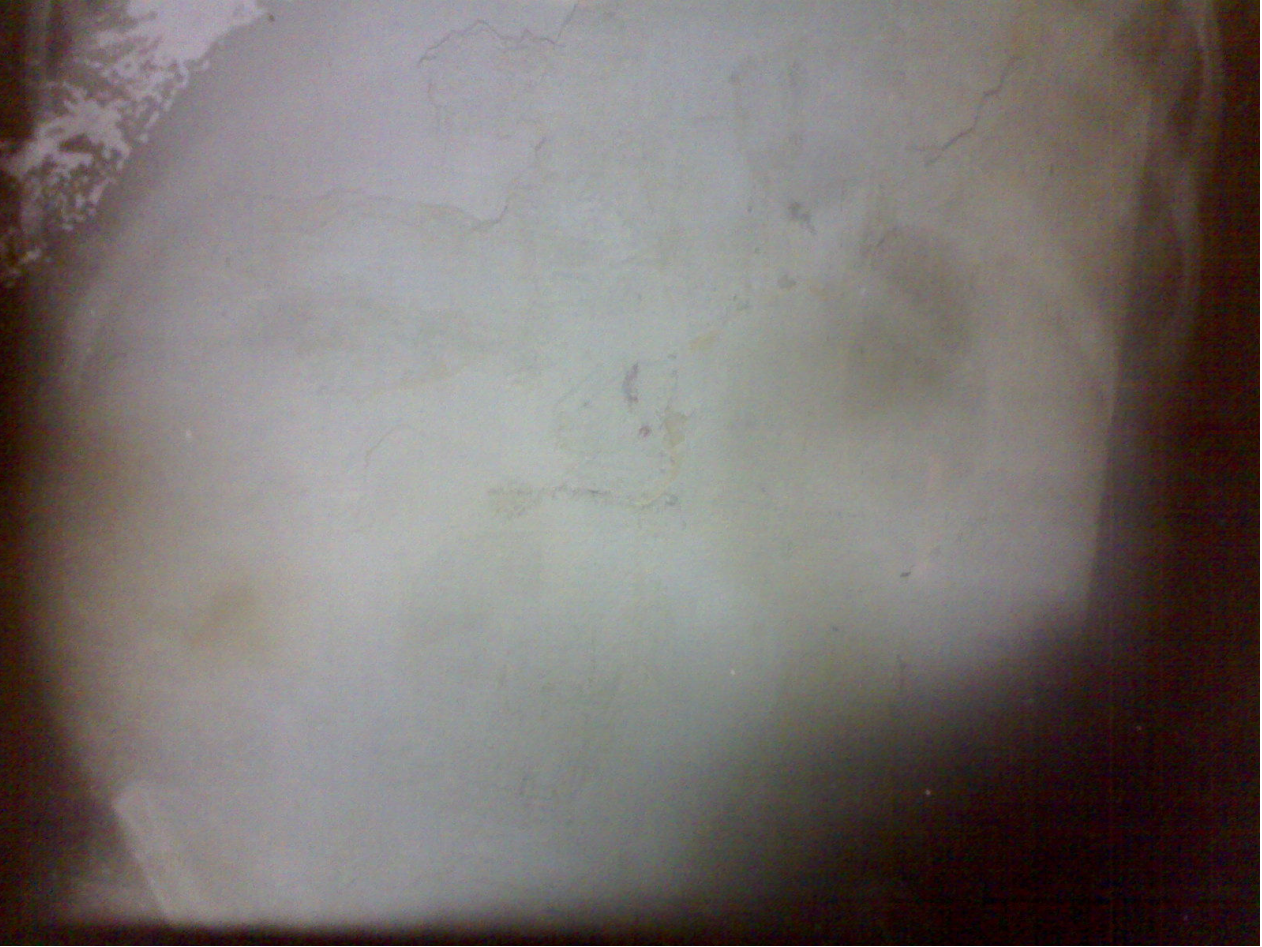
X-ray abdomen (erect) showing diffuse ground glass appearance.

X-ray abdomen erect showed diffuse ground glass appearance in the abdomen (Fig. 1). No air fluid levels no gas under diaphragm was seen.

Abdominal ultrasound showed intraperitoneal fluid collection.

Diagnostic peritoneal tapping revealed a non bloody straw colour fluid which was found to be urine. The collected urine was found to be sterile.

IVP or cystogram were not undertaken as the peritoneal tap and ultrasound confirmed the presence of urine in the abdomen, in case of doubt i definetely recommend these tests.

### Treatment

The patient was catherized and put on intravenous ceftriaxone and metronidazole pre-operatively and was continued on the same for a period of 5 days. Emergent exploratory laparatomy was performed; intraperitoneal rupture of the bladder as a result of scar dehiscence of the previous bladder rupture repair was found located on the dome of the bladder and was repaired in layers. About two litres of urine was drained out of the peritoneal cavity. The rest of the bladder was found to be absolutely normal. The girl's recovery was fast and uncomplicated; her blood urea and creatinine levels were in the normal range in just two days after the operation. Subsequently she was discharged on oral antibiotics and dietary supplements with advice for regular follow up.

## Discussion

The fore mentioned case is only the third to be reported till date, the first one being in 1992 in UK [1]. The second one was in 2004 in Nepal [3]. Henceforth spontaneous scar dehiscence of a bladder scar seems to be very rare, most of the other reported cases have had some accompanying pathology involved. Diagnosing bladder rupture is not very difficult if a watchful eye is kept on the signs and symptoms, imaging techniques are of great help. A cystogram or a diagnostic peritoneal tap can lead to a confirmatory diagnosis. Intra-peritoneal rupture of the urinary bladder is about six times as common as that of extra-peritoneal rupture of the bladder. Leaking of urine in the peritoneal cavity in case of intra peritoneal rupture leads to absorption of urea and urinary products from the gut leading to increase in the patients blood urea and creatinine, which essentially comes back to normal after the repair. Conservative treatment of bladder rupture has an increased chance of recurrence and so do have procedures like partial cystectomy, in recurrent cases augmentation cystoplasty or urinary diversion should be considered as the treatment.

Studying at all the three documented cases closely brings out some interesting facts, to enumerate firstly in all the three documented cases spontaneous bladder scar ruptures occurred about 3 to 4 years after the original bladder repair was carried out, in the earlier two cases exactly after 3 yrs, my case had the greatest time gap-that of 4 years. Secondly both my case and that reported in Nepal involved children (I don't have the details of the one reported in UK). In my view as bladder is subjected to tremendous pressure variations throughout the day, spontaneous scar dehiscence is likely as a result. It can be pointed out that children are on an even higher risk because because they are not as "toilet trained" as adults are, especially children who avoid voiding and hold urine for long periods of time. The above observations reinforce the need for proper patient education and that of regular follow up in cases of bladder rupture, at-least up to a minimum of 5 years after the primary repair procedure is carried out and more so in cases of bladder rupture involving children.

## Conclusion

It seems that bladder scar dehiscence is more common in children and that too within 4 yrs of the primary repair. Bladder rupture can be easily diagnosed early with a detailed history and imaging techniques like USG and AXR, confirmed by a cystogram or diagnostic peritoneal tapping. Most of the cases are not infected but should be put on IV antibiotics nevertheless. A surgical repair or the bladder should be done immediately on confirmation instead of trying to manage the case conservatively. Augmentation cystoplasty or urinary diversion should be considered in recurrent cases. I would also like to emphasize the need of proper education and toilet training for children as well as regular follow up after a primary bladder rupture repair, more so in the beginning 3 to 4 yrs of surgery.

## Consent

Written informed consent was obtained from the patient for publication of this case report and accompanying images. A copy of the written consent is available for review by the Editor-in-Chief of this journal.

## Competing interests

The author declares that they have no competing interests.

## Authors' contributions

AS collected, analyzed and interpreted the patient data regarding the scar dehiscence of a previous repaired urinary bladder rupture. AS has been involved in drafting the manuscript or revising and has given final approval of the version to be published.
